# Understanding the Lower and Upper Limits of Sample Sizes in Clinical Research

**DOI:** 10.7759/cureus.76724

**Published:** 2025-01-01

**Authors:** Abdullah A Hamad, Sirwan K Ahmed

**Affiliations:** 1 Faculty of Medicine, Menoufia University, Shibin El-Kom, EGY; 2 Medical Research Group of Egypt, Negida Academy, Arlingon, USA; 3 College of Nursing, University of Raparin, Rania, IRQ

**Keywords:** clinical research, education, reliability, research method, sample size, sample size and power computation, sample size estimation, sample size formula, sampling, validity

## Abstract

The determination of an appropriate sample size is pivotal in medical research, not only for achieving statistical adequacy but also for ensuring ethical integrity and resource efficiency. This editorial elucidates the complexities involved in defining the lower and upper limits of sample size across various research paradigms. The lower limit is essential for maintaining sufficient statistical power and precision, which depend on several factors, including the study's objectives, inherent population variability, and the desired accuracy of results. An insufficient sample size poses a risk of significant Type II errors and produces wide confidence intervals, thereby undermining the reliability and applicability of the research findings. Conversely, the upper limit encounters practical constraints related to resource allocation and ethical considerations, where the principle of diminishing returns becomes evident as sample sizes increase beyond a certain threshold. This scenario leads to minimal gains in precision at the cost of potential participant risk and resource overutilization. The editorial advocates for a methodical approach to sample size calculation, utilizing statistical tools such as sample size formulas, and G*Power and adopting innovative methodologies, including adaptive trial designs and Bayesian statistics. These strategies facilitate dynamic adjustments based on interim results and prior knowledge, respectively, promoting optimal resource utilization while preserving robust statistical power. Ultimately, the careful calibration of sample size enhances the validity and ethical integrity of medical research, thereby bolstering its contribution to scientific knowledge.

## Editorial

In medical research, studying an entire population is often impractical. To address this, researchers rely on samples that aim to represent the population and allow for the generalization of findings [1]. Determining the appropriate sample size is a critical component of study design. A sample that is too small may fail to detect significant effects, while an excessively large sample can result in wasted resources and ethical challenges. Understanding the concepts of upper and lower limits of sample size is essential for conducting ethical, efficient, and scientifically robust research.

The lower limit of sample size ensures adequate statistical power and precision. However, there is no universal minimum sample size that applies to all studies. The appropriate lower limit depends on factors such as study objectives, population variability, and the desired level of precision [2]. A sample size that is too small can lead to significant limitations in research outcomes. Low statistical power reduces the ability to detect true effects, thereby increasing the risk of Type II errors (false negatives). Additionally, small sample sizes often produce wide confidence intervals, resulting in imprecise parameter estimates that limit the applicability and reliability of findings. Lastly, insufficient sample sizes increase the vulnerability of results to random error and potential bias, making conclusions less dependable. However, small sample sizes are sometimes necessary for specific studies, such as Phase 1 drug trials, where the primary goal is to assess initial safety rather than therapeutic effects, and formal sample size calculations may not be required.

Key factors to consider in determining an appropriate sample size include statistical power, Type I error, and Type II error [3]. Statistical power, typically set at 80% (0.8) in medical research, represents the probability of detecting a true effect when one exists. Type I error (α) is the risk of rejecting a true null hypothesis and is conventionally set at 5% (0.05), corresponding to a 95% confidence level. Type II error (β) is the risk of failing to reject a false null hypothesis and is inversely related to statistical power, calculated as β = 1 - power. Ensuring these parameters are balanced is critical for producing valid and reliable results. To assist in sample size calculation, researchers can use tools like the GPower software, which provides calculations based on study design and statistical tests, or apply sample size formulas that consider factors such as power, error rates, and expected effect size [2].

**Figure 1:**
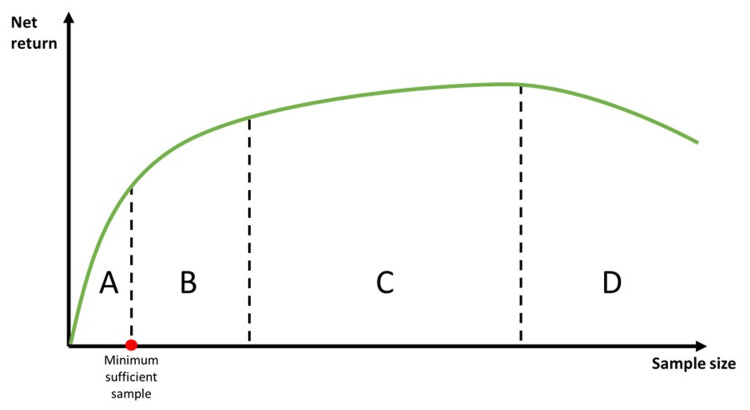
Demonstrates the relationship between sample size and return in research studies. In area A, sample sizes are insufficient, lacking the statistical power required to reliably detect the effect estimate, thereby increasing the risk of Type II errors. In area B, sample sizes achieve adequate statistical power, with further increases significantly improving precision. This may be necessary to address weak sampling methods, account for variability, or adjust for potential confounders. Beyond this lies area C, marking the *diminishing returns* threshold, where additional increases in sample size yield minimal improvements in the precision or accuracy of results. Finally, in area D, increasing the sample size further leads to negative returns due to disproportionate costs, time, and resource demands, along with ethical concerns about unnecessarily exposing additional participants to study procedures for only marginal gains in accuracy. Image credit: Abdullah A. Hamad.

In contrast to the focus on lower sample size limits, discussions on the upper limit are less common. Larger sample sizes generally enhance precision by minimizing sampling error, but practical, ethical, and economic considerations impose constraints. The principle of diminishing returns applies, where the marginal gain in precision decreases as the sample size grows. Beyond a certain point, additional participants contribute minimally to the accuracy of results [4]. Although this threshold is not a fixed point, it signifies a stage where the accuracy returns diminish progressively with oversampling (Figure 1). Resource constraints also play a significant role, as larger studies demand more time, funding, and personnel, potentially straining budgets and delaying results. Ethical considerations further underscore the need for restraint, as enrolling more participants than necessary exposes individuals to risks without adding meaningful scientific value. For example, in a hypertension drug trial, oversampling may expose participants to unnecessary side effects without improving the study's reliability, wasting resources and potentially harming vulnerable populations. Similarly, a small sample size that fails to detect a true effect is also considered unethical. This ethical dilemma emphasizes the importance of precise sample size calculation to ensure that research maximizes benefits while minimizing unnecessary harm and resource waste [5].

Moreover, oversampling in intervention studies can lead to the detection of statistically significant but clinically irrelevant differences between groups. This may overstate the intervention's effectiveness and diminish its applicability to real-world contexts. For instance, while larger samples reduce sampling error, they can also overinflate trivial differences, undermining the practical relevance of findings.

Therefore, determining the optimal sample size requires balancing statistical rigor, practicality, and ethical principles. While the lower limit ensures sufficient power and precision, the upper limit reflects the constraints of diminishing returns, resource availability, and participant welfare. Researchers should prioritize obtaining a sufficient, representative sample over maximizing participant numbers. Employing robust sampling techniques, such as stratified or random sampling, can further enhance the quality of research outcomes without unnecessary participant recruitment. Additionally, newer approaches such as adaptive trial designs and Bayesian statistics offer innovative ways to optimize sample size determination. Adaptive trial designs adjust the sample size dynamically based on interim results, allowing researchers to optimize resources and maintain statistical power. Bayesian statistics use prior knowledge to iteratively update sample size decisions as new data is collected, allowing for more efficient resource use.
